# Long-read transcriptomics corrects *Trichomonas vaginalis* intron annotations and refines transcript-end features

**DOI:** 10.1186/s13071-026-07530-x

**Published:** 2026-06-23

**Authors:** Yuan-Ming Yeh, Wei-Hung Cheng, Kuo-Yang Huang, Chi-Ching Lee, Seow-Chin Ong, Hong-Wei Luo, Jhen-Wei Syu, Cheng-Hsun Chiu, Po-Jung Huang, Petrus Tang

**Affiliations:** 1 Genomic Medicine Core Laboratory, Chang Gung Memorial Hospital, Linkou, Taoyuan City, Taiwan; 2 Chang Gung Microbiota Therapy Center, Chang Gung Memorial Hospital, Linkou, Taoyuan City, Taiwan; 3 Graduate Institute of Health Industry Technology, Chang Gung University of Science and Technology, Taoyuan City, Taiwan; 4 Department of Microbiology and Immunology, College of Medicine, National Cheng Kung University, Tainan City, Taiwan; 5 Department of Parasitology, College of Medicine, National Cheng Kung University, Tainan City, Taiwan; 6 Host-Parasite Interactions Laboratory, National Defense Medical Center, Taipei City, Taiwan; 7 Department of Computer Science and Information Engineering, College of Engineering, Chang Gung University, Taoyuan City, Taiwan; 8 Department of Parasitology, College of Medicine, Chang Gung University, No. 259, Wenhua 1st Rd., Guishan Dist., Taoyuan City, 333 Taiwan; 9 Molecular Infectious Disease Research Center, Chang Gung Memorial Hospital, Linkou, Taoyuan City, Taiwan; 10 Department of Biomedical Sciences, College of Medicine, Chang Gung University, No. 259, Wenhua 1st Rd., Guishan Dist., Taoyuan City, 333 Taiwan; 11 Molecular Medicine Research Center, Chang Gung University, Taoyuan City, Taiwan

**Keywords:** *Trichomonas vaginalis*, Long-read transcriptomics, Direct RNA sequencing, Intron annotation, Polyadenylation, 5′/3′ UTR, UAAA signal, Genome annotation

## Abstract

**Background:**

*Trichomonas vaginalis* causes the most prevalent non-viral sexually transmitted infection worldwide. Despite its large genome (181.5 Mb; 36,310 predicted protein-coding genes in NYU_TvagG3_2), intron annotations remain limited and inconsistently validated. A recent short-read RNA-seq study reported 63 putative active introns, but short reads can misassign splice boundaries and cannot resolve complete transcript structures.

**Methods:**

We integrated Oxford Nanopore direct RNA sequencing (DRS), ONT cDNA long-read sequencing, and Illumina RNA-seq to refine intron annotations, transcript-end features, and UTR boundaries in *T. vaginalis*. Candidate introns were validated by targeted PCR and Sanger sequencing, and representative splicing events were further assessed using public SRA datasets.

**Results:**

Starting from 31 historically annotated introns, motif-guided long-read screening and orthogonal validation identified 17 additional validated introns, increasing the curated set to 48 confirmed introns. Among these 17 events, three were previously unrecognized in the current NYU_TvagG3_2 reference annotation. We also corrected five reported loci, including two false-positive introns, two splice-coordinate misannotations, and one gene-sequence error. DRS further supported transcript termination site mapping, UAAA polyadenylation-signal profiling relative to poly(A) addition sites, and single-molecule poly(A)-tail estimation. StringTie mixed-mode assemblies provided updated UTR boundaries for intron-bearing transcripts and transcripts without curated introns.

**Conclusions:**

This study provides a rigorously validated, long-read-refined resource of intron annotations, UTR boundaries, and UAAA-guided transcript-end features for *T. vaginalis*, together with a reproducible workflow for non-model protists. These refinements improve the current reference annotation and support future studies of functional genomics, parasite biology, pathogenesis, and diagnostic development.

**Graphical Abstract:**

**Figure:**
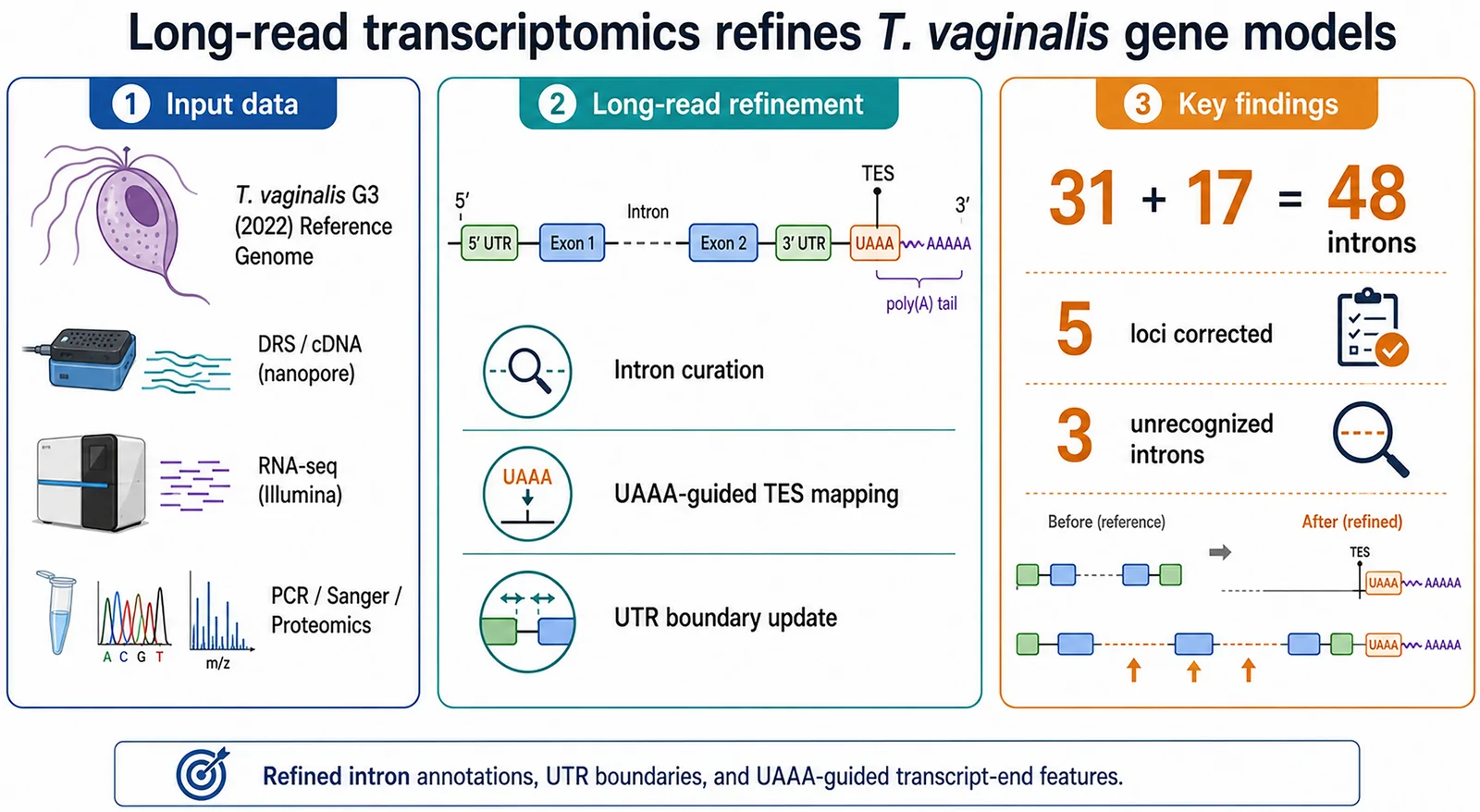

**Supplementary Information:**

The online version contains supplementary material available at https://doi.org/10.1186/s13071-026-07530-x.

## Background

Sexually transmitted infections (STIs) remain a major global public-health challenge. According to the World Health Organization, more than one million curable STIs are acquired each day, accounting for hundreds of millions of new infections annually caused by *Chlamydia trachomatis, Neisseria gonorrhoeae*, *Treponema pallidum*, and *Trichomonas vaginalis*, with trichomoniasis representing the largest proportion of cases [1–3].

*Trichomonas vaginalis* propagates as motile, pear-shaped trophozoites that adhere to vaginal epithelial cells and can transition into amoeboid forms that promote host-tissue damage. In addition to this morphological plasticity, cyst-like "pseudocyst" forms characterized by internalized axonemes and flagella have also been described, suggesting the presence of alternative survival strategies that likely depend on extensive transcriptional reprogramming in response to fluctuating microenvironmental conditions [4–6].

*T. vaginalis* possesses one of the largest known protist genomes. The G3 reference assembly published in 2007 revealed a genome exceeding 160 Mb in size, characterized by a highly repetitive architecture (~ 65% repetitive content) and extensive gene duplication [7–10]. A chromosome-scale reassembly of *T. vaginalis* was subsequently generated using long-read sequencing in combination with Hi–C scaffolding. This effort produced six chromosome-scale scaffolds spanning 184.2 Mb, with substantially improved structural annotation encompassing approximately 37,794 predicted protein-coding genes and extensive transposable-element (TE) content, consistent with the published karyotype and fluorescence in situ hybridization (FISH)-mapped rDNA loci [11]. The corresponding NYU_TvagG3_2 reference genome, released in 2022, incorporated PacBio long-read data alongside Illumina and Hi–C sequencing data [12, 13], consistent with the reported reassembly strategy. Collectively, comparative genomic studies have highlighted extensive gene-family expansion and pervasive TE activity associated with host interaction, adaptation, and metabolic diversification [11, 14, 15].

Paradoxically, spliceosomal introns are extremely rare in the *T. vaginalis* genome. Early genomic surveys estimated an intron density of fewer than 0.001 introns per gene and identified two unconventional intron classes [7, 16–18]. Recently, a short-read RNA-seq study reported 63 "active" introns and 34 putative "inactive" intron-like sequences using an extended-motif filtering pipeline to distinguish authentic splicing events from chimeric alignments, together with PCR validation of newly predicted candidates. That study also described an exceptionally short 23-nt intron and an unexpected enrichment of introns within untranslated regions (UTRs). In addition, it assessed intron conservation using a chromosome-scale assembly of *Trichomonas stableri* [19]. However, the intrinsic read-length limitations of short-read sequencing can lead to inaccurate splice-boundary assignments and hinder precise annotation of UTRs and polyadenylation-related features. More importantly, short reads cannot phase complete transcript termini and, therefore, cannot determine whether an observed transcription start site (TSS) and poly(A) site originate from the same RNA molecule.

Here, we address these limitations using long-read transcriptomics while explicitly incorporating the experimentally validated *T. vaginalis*-specific UAAA polyadenylation signal [20, 21]. By integrating Oxford Nanopore direct RNA sequencing (DRS), ONT cDNA long-read sequencing, and Illumina RNA-seq, we refined intron annotations, reconciled and expanded the existing intron catalog, and generated long-read-supported annotations of 5′ and 3′ UTR boundaries, UAAA polyadenylation-signal positions relative to DRS-defined transcript-end sites, and single-molecule poly(A)-tail length distributions. Collectively, this long-read-corrected resource provides an improved reference framework for future functional genomics and diagnostic applications.

## Methods

### T. vaginalis culture

*Trichomonas vaginalis* strain ATCC PRA-98 (G3; ATCC) was maintained in yeast extract–iron–serum (YI-S) medium at pH 5.8, supplemented with 10% (v/v) heat-inactivated horse serum, 1% glucose, and 80 µM ferric ammonium citrate, at 37 °C under axenic conditions [22]. Mid-log trophozoites (1.5 × 10^6^ cells/mL) were harvested for all experiments. Viability was assessed by trypan blue exclusion.

### Nucleic acid extraction

Genomic DNA was extracted from ~ 5 × 10^6^ trophozoites using the QIAGEN Genomic-tip (Blood and Cell Culture DNA Kit) according to the manufacturer's instructions. Briefly, cells were lysed in Buffer QBT, applied to the Genomic-tip by gravity flow, washed with Buffer QC, and eluted with Buffer QF. DNA was precipitated with 0.6 volumes isopropanol at room temperature for 10 min and resuspended in nuclease-free water. Total RNA was isolated from mid-log cultures using the GENEzol TriRNA Pure Kit (Geneaid, Taiwan) per the manufacturer's protocol. First-strand cDNA for targeted validations was synthesized using SuperScript^™^ III (Invitrogen).

### Long- and short-read sequencing

For Oxford Nanopore direct RNA sequencing (DRS), libraries were prepared from 500 ng total RNA using the Direct RNA Sequencing kit SQK-RNA002 (Oxford Nanopore Technologies, ONT) without artificial poly(A) tailing. Library preparation followed the ONT protocol (oligo(dT) splinting, reverse transcription for strand stabilization, adaptor ligation), and libraries were sequenced on FLO-MIN106 (R9.4.1) flow cells on a MinION for up to 48 h using MinKNOW software (version 21.02.5, with core 4.2.11). Basecalling was performed with Guppy v6.0.6 using high-accuracy RNA models.

For ONT cDNA PCR sequencing, libraries were prepared with SQK-PCS109 (ONT) from total RNA according to the manufacturer's instructions and sequenced on FLO-MIN106 for up to 48 h. Basecalling was performed with Guppy v6.0.6 using high-accuracy DNA models.

For Illumina mRNA-seq, Poly(A)-enriched libraries were prepared from 200 ng total RNA using the Universal Plus mRNA-Seq kit with NuQuant (Tecan/NuGEN). After QC (Qubit dsDNA HS; Agilent Bioanalyzer High Sensitivity DNA), libraries were sequenced (2 × 150 bp) on an Illumina NovaSeq 6000, yielding ~ 66 M paired-end reads per sample.

All sequencing data generated in this study have been deposited in the NCBI Sequence Read Archive (SRA) under BioProject accession number PRJNA1311745.

### Public datasets

To increase replication and external validity, we re-analyzed public ONT DRS datasets reported by Sajek et al. (biological triplicates; SRR28202878–SRR28202880) using the same alignment and QC settings used in this study [23].

### Data processing and alignment

**Short reads.** Raw reads were quality-checked with fastp v0.20.1 [24] using default parameters. Reads were aligned to the *T. vaginalis* reference genome NYU_TvagG3_2 using HISAT2 v2.2.1 with splice-aware settings [25] for spliced alignments. Subsequent transcript assembly and quantification were performed using StringTie v2.2.1 [26, 27] and annotations from TrichDB/VEuPathDB release 65 (NYU_TvagG3_2) [28].

**Long reads.** DRS FASTQ and cDNA FASTQ reads (optionally error-corrected; see below) were aligned to NYU_TvagG3_2 using minimap2 v2.26 [29]. Parameters were optimized per molecule type: DRS reads were aligned using -ax splice -uf -k14 -G 10000 -L --secondary = no, whereas cDNA reads were aligned using -ax splice -k14 -G 10000 -L. Where needed, cDNA reads were hybrid-corrected with proovread v2.14.1 using Illumina short reads [30].

### Transcript assembly and quantification

Short- and long-read alignments were assembled and quantified with StringTie v2.2.1 using the NYU_TvagG3_2 reference annotation from TrichDB/VEuPathDB release 65 [26–28].

### Intron discovery and motif-guided search

We first extracted annotated multi-exon protein-coding loci from TrichDB/VEuPathDB release 65 (NYU_TvagG3_2) and curated 31 previously reported introns for completeness, intron length, splice-site sequences, and intron phase, as previously described [16–18]. To identify additional candidates, we performed a genome-wide regular-expression scan using revised splice-site motifs derived from these studies ('GYAYGYN{41,178}RCTAACACAYAG' and 'GTWYWDN{7}TCTAACH{1,2}AACAG'; Python-style regular expressions) and then required read-level support from both DRS and cDNA long reads at the junctions. Candidate introns were retained for downstream evaluation only when supported by splice-aware long-read alignments and validation evidence.

### UTR mapping and poly(A)-tail length estimation

Illumina RNA-seq, Oxford Nanopore direct RNA sequencing (DRS), and ONT cDNA reads were aligned to the *T. vaginalis* G3 genome (NYU_TvagG3_2). For polyadenylation signal (PAS) and poly(A)-site analyses, DRS alignment endpoints were converted into single-nucleotide transcript-end site (TES) records. Only reads with PASS poly(A) calls from Nanopolish were retained. TES positions were clustered independently by chromosome and strand using a 24-nt merging window, and the median coordinate of each cluster was used as the representative TES. Poly(A)-tail lengths were estimated directly from raw DRS current signals using Nanopolish PolyA v0.14.0 and summarized at both the gene level and by transcript class, including intron-containing genes and genes without curated introns [31]. For each TES cluster, the 0–100 nt upstream transcript-oriented reference sequence was scanned for the experimentally validated UAAA polyadenylation signal, implemented as the DNA motif TAAA [20, 21]. When multiple motif occurrences were present, the nearest upstream motif was retained. PAS-to-TES distance was defined as the upstream distance from the 5′ boundary of the TAAA motif to the TES coordinate and was reported as a non-negative value within the scanned 0–100 nt upstream window.

UTR differences were calculated from long-read transcript endpoints as ΔUTR = (long-read UTR length) − (reference UTR length). For 5′-end analyses, Illumina RNA-seq, DRS, and ONT cDNA alignments were jointly assembled using StringTie v2.2.1 in mixed mode with reference-guided anchoring (-G), and the representative transcription start site was defined as the highest-TPM isoform per gene. For 3′-end analyses, LRmix TES cluster tables were compared against reference annotations using the dominant supported TES cluster per transcript. Genes lacking reliable long-read or reference-supported endpoints were excluded for the corresponding end-specific analyses. Differences within ± 10 nt were considered unchanged. Sensitivity analyses were performed using TPM thresholds of ≥ 1 and ≥ 5. The intron-containing gene set was defined from the curated intron list, and all remaining annotated genes were classified as genes without curated introns for group comparisons. Group differences were assessed using two-sided Mann–Whitney *U* tests, with effect sizes reported as Cliff’s δ and bias-corrected and accelerated (BCa) bootstrap 95% confidence intervals (2,000 resamples). Fisher’s exact tests were used for proportion comparisons at defined |Δ| thresholds.

Data visualization included violin plots (showing means, medians, and extrema; the *y*-axis displayed ± 300 nt or was winsorized solely for visualization purposes) and empirical cumulative distribution function (ECDF) plots; all statistical analyses were performed using raw, untransformed values. Analyses were conducted in Python using pandas, numpy, scipy, and matplotlib, and all source tables and figure panels are provided with the manuscript.

### Functional annotation (newly identified intron-containing genes)

Open reading frames (ORFs) were predicted and translated for downstream BLASTp analyses. InterProScan v5.60-92.0 [32] was used with default parameters to identify protein domains and functional sites by searching against Pfam 35.0 [33] and the Conserved Domain Database (CDD) 3.20 [34] to annotate domains and functional sites. Automated domain annotations were subsequently curated through manual inspection of homologous sequences across related taxa to ensure functional consistency and to reduce annotation artifacts. Short-read and long-read alignments were visualized using IGV v2.16.0 [35] for manual inspection of exon–intron structures, splice-junction support, and UTR boundaries.

### PCR validation of introns

Primers flanking predicted introns, with at least one primer spanning the exon–exon junction where applicable, were designed using Primer3 [36] (Table S5). PCR assays were performed using genomic DNA (gDNA) and cDNA templates, with no-template controls (NTCs) and –RT controls included in each experiment. Reactions were carried out using 2 × Super Hi-Fi Taq PCR MasterMix containing loading dye (KTT-BB05, BIOTOOLS Co.). Amplified products were excised, purified, and subjected to Sanger sequencing. Resulting sequences were aligned to the reference genome to confirm exon–exon junctions and precisely define intron boundaries.

### Proteomic analysis

Total protein was extracted from *T. vaginalis* trophozoites, digested with trypsin, and analyzed by LC–MS/MS using a Q Exactive Orbitrap mass spectrometer (Thermo Fisher Scientific). To specifically validate transcriptomic predictions, we constructed a custom protein database by translating the open reading frames (ORFs) of the 17 intron-containing gene models refined in this study. This targeted strategy was selected over the broader set of 63 previously reported introns [19], as many of those models lacked complete ORF definitions required for confident peptide-spectrum matching. MS/MS spectra were searched against this custom database, appended to the standard *T. vaginalis* reference proteome, using Proteome Discoverer (v2.4.1.15). Peptide and protein identifications were filtered at a 1% false discovery rate (FDR).

**Fig. 1:**
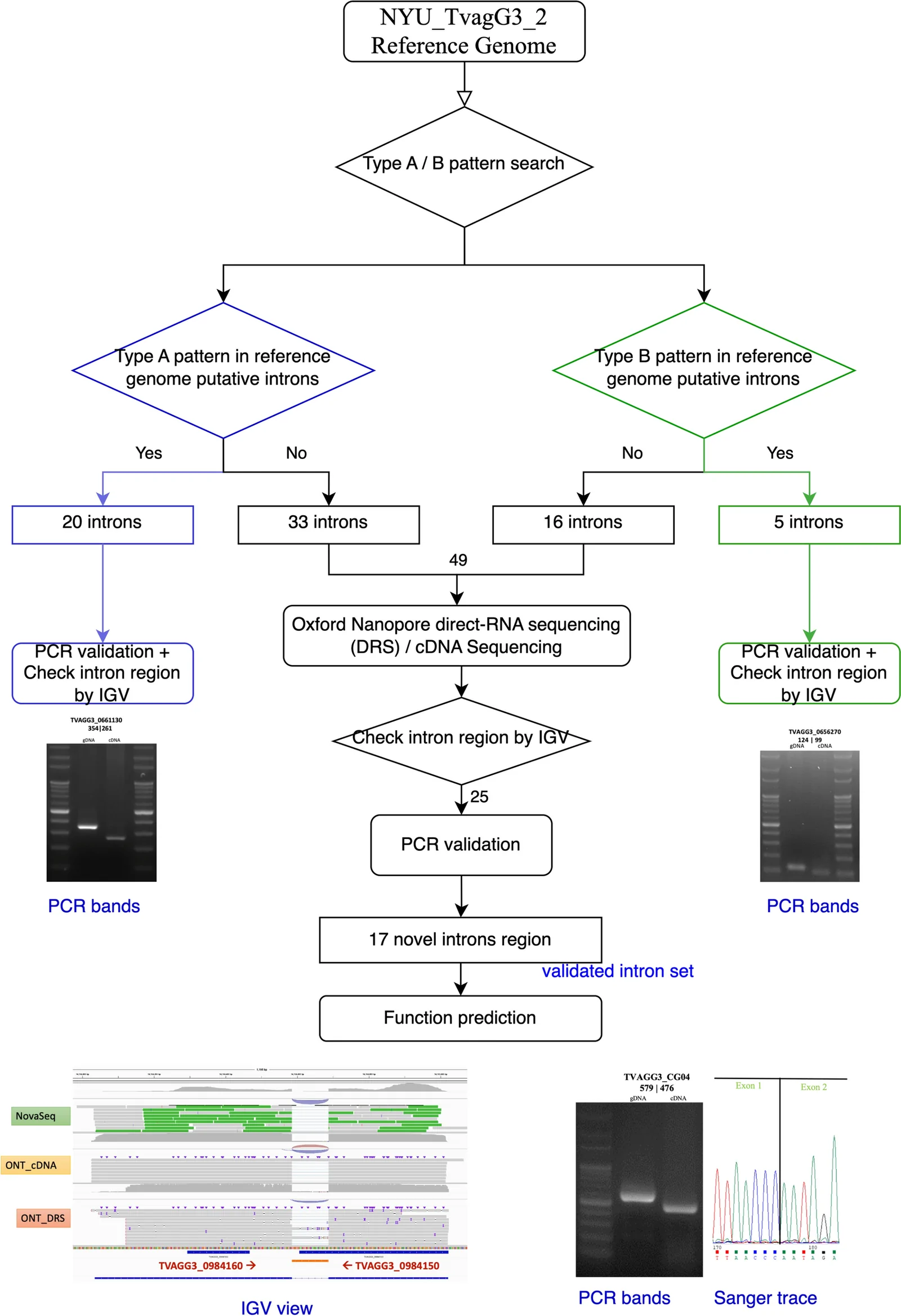
Integrated bioinformatics discovery and experimental validation workflow for intron-containing genes in *Trichomonas vaginalis*. Putative introns were classified as Type A or Type B based on motif patterns searched across the NYU_TvagG3_2 genome. Historically annotated Type A (*n* = 20) and Type B (*n* = 5) introns were subjected to PCR validation, whereas additional motif-supported candidate loci (*n* = 49) were examined using Oxford Nanopore long-read datasets, including direct RNA sequencing (DRS) and cDNA sequencing. IGV inspection shortlisted 25 candidates with long-read splice-junction support, of which 17 were validated by PCR, with representative events further confirmed by Sanger sequencing. Representative gel electrophoresis images, IGV screenshots, and Sanger traces are shown

The complete bioinformatics workflow, including read processing, hybrid assembly, and motif-guided intron discovery, is summarized in Fig. 1.

## Results

### Challenges posed by a highly repetitive genome

The genome of *Trichomonas vaginalis* is dominated by transposable-element (TE) families and large multicopy gene families, resulting in extensive sequence redundancy. Combined alignments of Oxford Nanopore direct RNA sequencing (DRS) and cDNA long reads initially identified 1,658 putative chimeric splice-junction transcripts, many spanning kilobase-to-megabase-scale genomic distances. Such mapping artifacts are a well-recognized but still incompletely resolved issue in repeat-rich genomes [37]. We initially attempted to validate a subset of multi-intron candidates (≥ 2 introns per gene) using PCR on both genomic DNA and cDNA; however, none of these predictions could be experimentally confirmed. This outcome prompted a redesign of the discovery pipeline, prioritizing motif-supported intron calls integrated with read-level evidence and orthogonal validation by PCR and Sanger sequencing (Fig. 1).

### Summary of consolidated intron‑containing genes

Using the chromosome-level reference *T. vaginalis* G3 (NYU_TvagG3_2), we first curated 31 historically annotated introns and classified them according to strict splice-site motifs (Type A and Type B) versus unassigned introns (lacking canonical motifs), based on previously reported patterns [16–18]. Of these, 25 introns (20 Type A and 5 Type B) showed clear read-level support in IGV from both DRS and ONT cDNA datasets and were PCR-positive in genomic DNA and cDNA assays. The remaining six annotated introns were classified as motif-unassigned; all were PCR-positive, confirming genomic presence. However, two of these lacked detectable expression in public DRS replicates (SRR28202878–SRR28202880) and were, therefore, inferred to be transcriptionally silent under the tested culture conditions, although their genomic integrity was verified by PCR (Table S1; Figs. S1 and S2). DRS data further enabled single-molecule poly(A)-tail length estimation for 15 of the 31 intron-containing genes, with a mean length of 69.0 ± 18.2 nt. For the remaining genes, low read depth or background signal precluded robust poly(A)-tail estimation (Table S1).

### Motif-guided discovery of additional introns

**Fig. 2:**
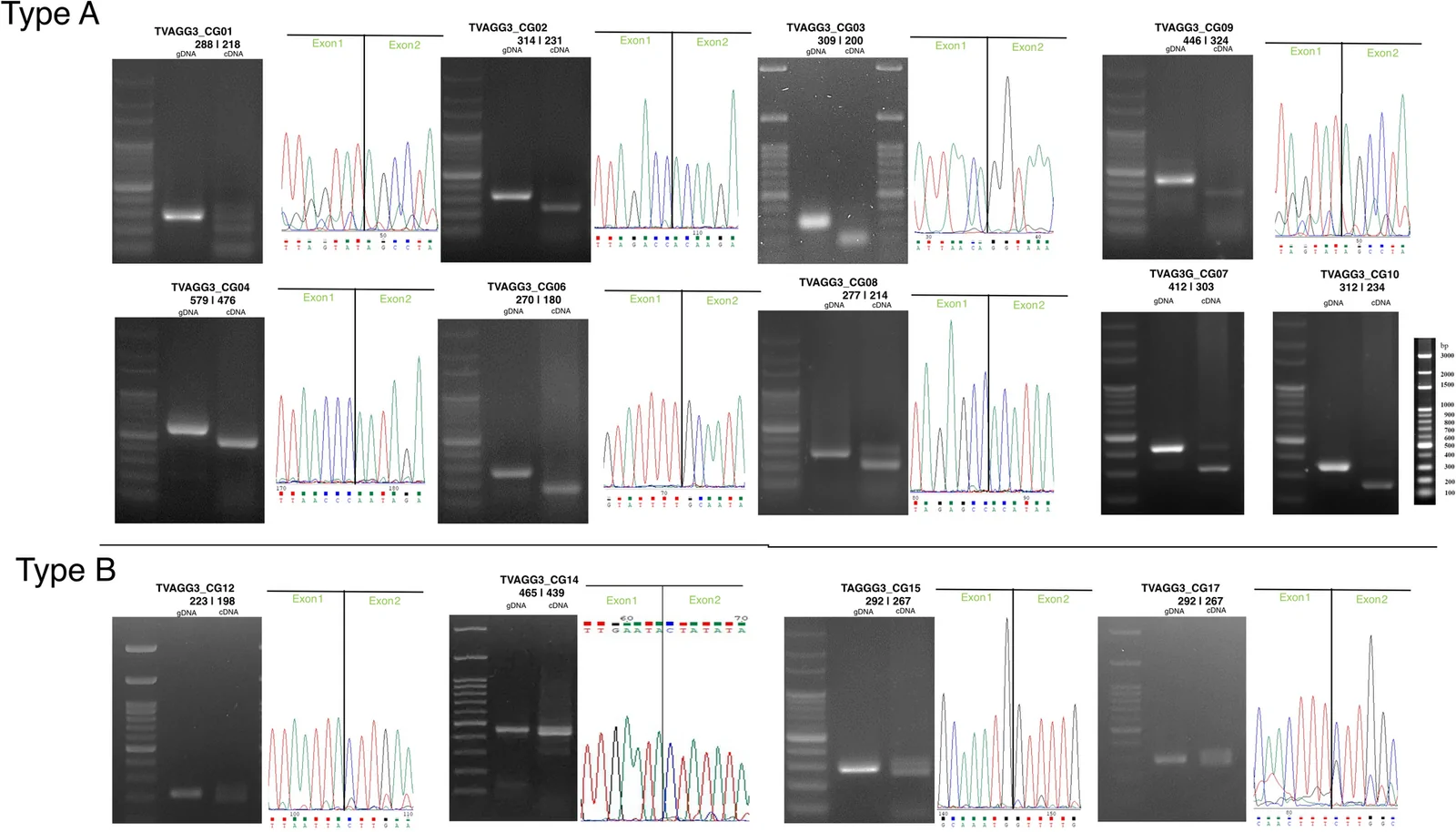
Validation and characterization of a representative subset of newly validated intron-containing genes in *Trichomonas vaginalis*. The figure shows 13 representative validated candidates, including nine Type A introns and four Type B introns, selected from the full set of 17 additional validated introns summarized in Table S2. PCR validation compared genomic DNA (gDNA) and cDNA templates to confirm intron-containing loci, with distinct amplicon-size differences supporting splicing. Sanger sequencing further verified exon–exon junctions and refined intron boundaries for representative events. Complete validation status for all 17 additional validated introns is provided in Table S2

We next scanned the NYU_TvagG3_2 reference genome of *T. vaginalis* G3 using stringent Type A (GYAYGYN{41,178}RCTAACACAYAG) and Type B (GTWYWDN{7}TCTAACH{1,2}AACAG) splice-site motifs, followed by relaxed motif variants (see Methods). This search identified 49 additional candidate introns (33 Type A-like and 16 Type B-like). Candidate validation required two criteria: (i) long-read splice-junction support in IGV from corrected DRS and ONT cDNA datasets and (ii) PCR amplification from both genomic DNA and cDNA. Twenty-five candidates satisfied the long-read support criterion, and 17 of these yielded expected PCR amplicons. A representative subset was further confirmed by Sanger sequencing to verify exon–exon junctions (Figs. 2, S3). The validated set comprises 11 Type A and 6 Type B introns (Table S2), increasing the curated set from 31 historically annotated introns to 48 confirmed introns.

### Provenance of the 17 additional validated introns

The 17 additional validated introns fell into five provenance categories (examples summarized in Table S2): (1) restored introns from earlier gene splits, in which loci were intron-bearing in earlier assemblies but lacked annotated introns in NYU_TvagG3_2 (TVAGG3_CG07; TVAGG3_CG10); (2) previously intron-naïve genes, representing introns discovered in genes not previously annotated as spliced (TVAGG3_CG03; TVAGG3_CG04); (3) 5′-UTR introns, located within untranslated regions (TVAGG3_CG05, _CG06, _CG08, _CG12, _CG13, _CG16, _CG17); (4) gene merges, in which introns span two previously annotated loci, suggesting gene-model fragmentation (TVAGG3_CG01, _CG02, _CG11, _CG14); and (5) novel loci, corresponding to introns within genes absent from the current annotation and assigned provisional identifiers pending stable naming (TVAGG3_CG09; TVAGG3_CG15). Thus, the 17 additional validated introns include events restored from earlier annotations, events that refine fragmented or incomplete gene models, and three introns previously unrecognized in the current NYU_TvagG3_2 reference annotation. Collectively, incorporation of these validated introns increased the total number of confirmed introns from 31 to 48, refined multiple gene models, and revealed a higher-than-expected proportion of UTR-localized introns, an architectural feature likely under-detected in short-read-based annotations (Tables 1, S2).

**Table 1:** Intron metrics and sequence features of the 17 additional validated intron-containing loci

	Gene ID	Predicted function	Intron length (bp)	GC content (%) (exon/intron)	IP^a^	RIP^b^	Phase	Exon–exon sequence (nucleotide)	Exon/exon amino-acid sequence^c^
Type A	TVAGG3_CG01	Transcription-initiator DNA-binding domain IBD	83	43.4/25.3	132	0.09	2	GACC/ACAA	IIR/pQE
	TVAGG3_CG02	Transcription-initiator DNA-binding domain IBD	102	38.8/25.5	104	0.12	2	ACCC/AATA	CIN/pIE
	TVAGG3_CG03	Protein kinase-like (PK-like)	109	37.5/12.8	204	0.07	1	AACA/GGTA	LPF/qDP
	TVAGG3_CG04	Transcription-initiator DNA-binding domain IBD	63	38.2/34.9	91	0.08	2	AGCC/ACAT	LIE/pHN
	TVAGG3_CG05	Unknown	60	33.2/33.3	47	0.03	0	TAAT/TTTG	LCN/FVV
	TVAGG3_CG06	Zinc finger DHHC domain-containing protein	90	30.7/18.9	186	0.08	2	TTGC/AAAA	RRI/aKY
	TVAGG3_CG07	CAMK-family protein kinase	114	39.4/24.6	200	0.07	0	TGCA/GGAT	LVA/GYL
	TVAGG3_CG08	CENTAURIN/ARF family	122	36.6/18.0	179	0.06	0	AACA/TTAT	TAT/LLM
	TVAGG3_CG09	Protein kinase-like (PK-like)	70	30.9/21.4	222	0.06	1	GGCT/ATAC	DLG/yTN
	TVAGG3_CG10	Predicted poly(A) polymerase	94	38.6/22.3	151	0.06	0	CGAA/ATTG	GIE/IDL
	TVAGG3_CG11	CENTAURIN/ARF family	116	37.5/24.1	127	0.06	0	TACA/CTTT	TAT/LLI
Type B	TVAGG3_CG12	Unknown	25	31.8/24.0	42	0.02	2	ATTA/CTTG	LIN/yLK
	TVAGG3_CG13	Ribonuclease inhibitor	25	32.1/28.0	9	0.01	1	ATTA/ACAG	KVI/nSF
	TVAGG3_CG14	Unspecified product	25	32.3/28.0	57	0.02	2	AATA/CTAT	LVE/yYI
	TVAGG3_CG15	Unknown	25	31.0/20.0	37	0.05	1	AAAC/CATT	NPK/pFA
	TVAGG3_CG16	Sm-like ribonucleoprotein family	25	35.0/24.0	8	0.08	1	GGTC/AAGA	YVG/qEV
	TVAGG3_CG17	Armadillo (ARM) repeat-containing family (5′-UTR)	25	33.5/24.0	n.d	0.03	n.d	CTTT/CTTG	n.d

Gene-level and intron-level properties are shown, including predicted function based on InterProScan, intron length, exon/intron GC content, intron position within the open reading frame (IP), relative intron position (RIP), intron phase, and exon–exon junction sequences at the nucleotide and amino-acid levels

^a^IP (intron position): position of the intron 5′ splice site relative to the first ATG, expressed in amino-acid coordinates

^b^RIP (relative intron position): IP divided by the total ORF length

^c^Lower-case amino acids indicate residues interrupted by a phase-1 or phase-2 intron. n.d., not determined; values were not determined because of ambiguous sequences, incomplete CDS, or localization within an untranslated region (e.g., 5′-UTR)

### Updated consensus motifs and sequence features

**Fig. 3:**
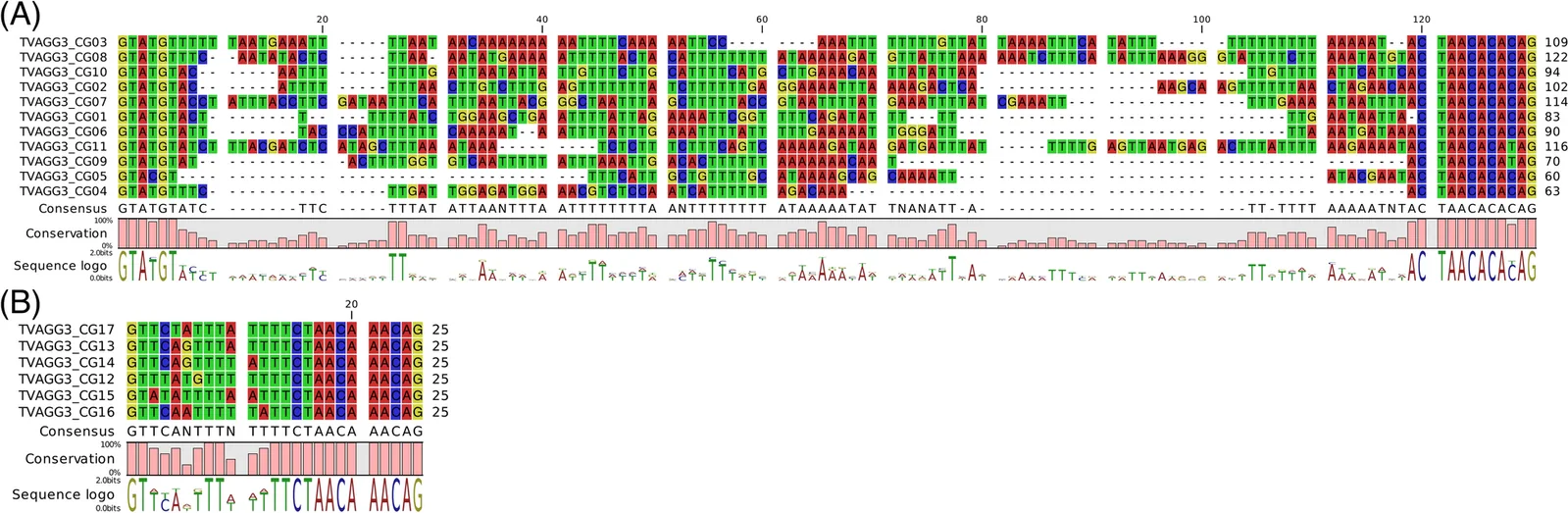
Multiple sequence alignment and sequence logos of newly validated introns. **A** Multiple sequence alignment of the 11 Type A introns identified in this study. **B** Alignment of the 6 Type B introns. The sequence conservation bar and sequence logo highlight nucleotide conservation across each intron class. Consensus donor (5′) and acceptor (3′) sites are indicated in bold. Numbers above alignment columns denote nucleotide positions; color coding follows the ClustalX convention (A = green, T = red, G = orange, C = blue)

Sequence features of the 17 introns are summarized in Tables 1 and 2 and visualized in Fig. 3. The refined consensus motifs derived from these validated events are as follows: Type A: GTAYGT{42,104}ACTAACACAYAG, with a tightened donor–acceptor spacing range relative to prior reports; and Type B: GTWYWD{7}TCTAACAAACAG. We additionally report intron phase distribution, GC content, and genomic localization (5′-UTR versus coding sequence) (Table 1), highlighting that several short introns are enriched in 5′-UTRs, where they may be linked to transcript-end features.

**Table 2:** Splice-site motifs of the newly validated Type A and Type B introns

Intron by Vanacova et al	5′-|GYAYGY	---(41–178 nt)---	ACTAACACAYAG|-3′
TVAGG3_CG01	5′-|GTATGT	----(65 nt)----	ACTAACACACAG|-3′
TVAGG3_CG02	5′-|GTATGT	----(84 nt)----	ACTAACACACAG|-3′
TVAGG3_CG03	5′-|GTATGT	----(91 nt)----	ACTAACACACAG|-3′
TVAGG3_CG04	5′-|GTATGT	----(45 nt)----	ACTAACACACAG|-3′
TVAGG3_CG05	5′-|GTACGT	----(42 nt)----	ACTAACACACAG|-3′
TVAGG3_CG06	5′-|GTATGT	----(72 nt)----	ACTAACACACAG|-3′
TVAGG3_CG07	5′-|GTATGT	----(96 nt)----	ACTAACACACAG|-3′
TVAGG3_CG08	5′-|GTATGT	----(104 nt)----	ACTAACACACAG|-3′
TVAGG3_CG09	5′-|GTATGT	----(52 nt)----	ACTAACACATAG|-3′
TVAGG3_CG10	5′-|GTATGT	----(76 nt)----	ACTAACACACAG|-3′
TVAGG3_CG11	5′-|GTATGT	----(98 nt)----	ACTAACACATAG|-3′
**Type A intron, consensus**	5′-|**GTAYGT**	---(42–104 nt)---	**ACTAACACAYAG**|-3′
*TvRab1a* intron by Deng et al	5′-|GTATAA	----(7 nt)----	TCTAACAAACAG|-3′
TVAGG3_CG12	5′-|GTTTAT	GTTTTTT (7 nt)	TCTAACAAACAG|-3′
TVAGG3_CG13	5′-|GTTCAG	TTTATTT (7 nt)	TCTAACAAACAG|-3′
TVAGG3_CG14	5′-|GTTCAG	TTTTATT (7 nt)	TCTAACAAACAG|-3′
TVAGG3_CG15	5′-|GTATAT	TTTAATT (7 nt)	TCTAACAAACAG|-3′
TVAGG3_CG16	5′-|GTTCAA	TTTTTAT (7 nt)	TCTAACAAACAG|-3′
TVAGG3_CG17	5′-|GTTCTA	TTTATTT (7 nt)	TCTAACAAACAG|-3′
**Type B intron, consensus**	5′-|**GTWYWD**	----(7 nt)----	**TCTAACAAACAG**|-3′

Consensus 5′ donor and 3′ acceptor splice-site sequences for each validated intron are aligned against previously reported motifs, including Type A introns reported by Vanacova et al. and the Type B TvRab1a intron reported by Deng et al. Dashes indicate intron-body length in nucleotides. Bold sequences indicate the refined consensus motifs derived from this study

**Y** = C or T; **W** = A or T; **D** = A, G, or T

### Functional updates from long-read-corrected models

Long-read evidence combined with PCR validation enabled structural and functional curation of several loci (details in Table S2). For example, TVAGG3_CG01/CG02 correspond to adjacent models that were merged into a single gene, with InterProScan supporting IBD family/transcription-initiator DNA-binding domains. TVAGG3_CG03/CG07/CG09 represent protein kinase-like loci, with CG07 re-annotated as a CAMK-family kinase. Splicing was experimentally confirmed for TVAGG3_CG10, annotated as a poly(A) polymerase-encoding locus, while merging of TVAGG3_CG11 supported a CENTAURIN/ARF-family annotation.

In addition, 5′-UTR extensions identified in TVAGG3_CG05, CG12, CG13, and CG17 introduce N-terminal peptide extensions that may alter predicted domain architectures. Importantly, long-read transcriptomics together with Sanger sequencing resolved a major sequence-level error (insertion/deletion) in the reference genome at the TVAGG3_CG03 locus of *T. vaginalis* G3. This error had previously led to incorrect automated annotations and short-read-based predictions of two truncated open reading frames. These data correct this discrepancy, restoring a single continuous coding sequence for this protein kinase-like locus (Fig. 5).

### Transcript-end refinement around intron-containing transcripts

**Fig. 4:**
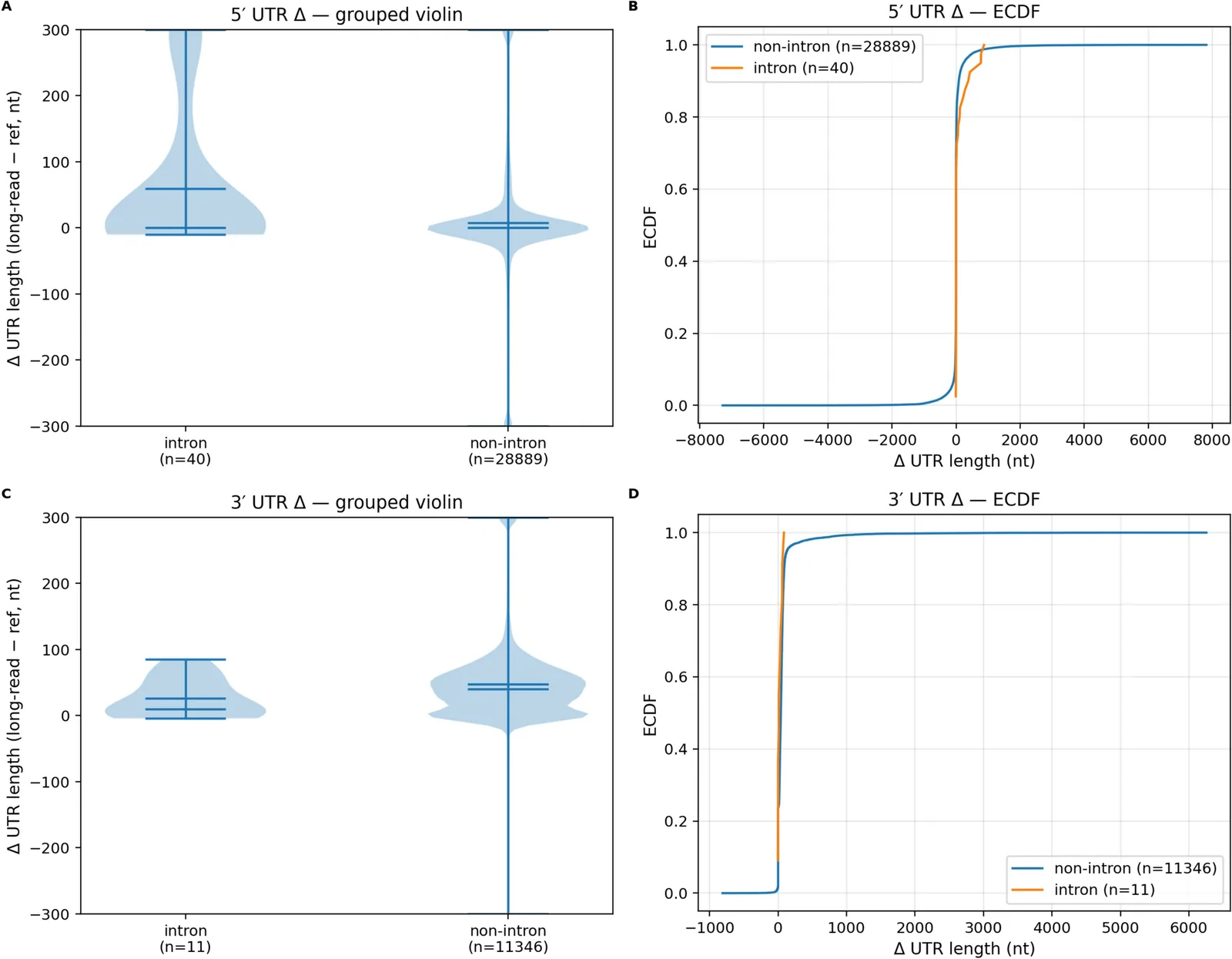
Long-read refinement of UTR boundaries in intron-containing genes and other genes. **A** 5′-UTR length differences (ΔUTR = long-read UTR length − reference UTR length) shown as violin plots for intron-containing genes (*n* = 40) and genes without curated introns (*n* = 28,889). Means, medians, and extrema are indicated. For display only, the *y*-axis is limited to ± 300 nt; statistical tests used raw values. The groups differed significantly (Mann–Whitney *U* *P* = 0.0052), with a small-to-moderate effect size (Cliff’s δ ≈ 0.25; BCa 95% CI 0.10–0.40), indicating greater 5′-UTR extension among intron-containing genes. **B** Empirical cumulative distribution functions (ECDFs) for the same 5′-UTR ΔUTR values, showing the right-shift of intron-containing genes. **C** 3′-UTR ΔUTR violin plots for intron-containing genes (*n* = 11) and genes without curated introns (*n* = 11,346). For display only, the *y*-axis is limited to ± 300 nt; statistical tests used raw values. The group difference was not significant (Mann–Whitney *U* *P* = 0.125; Cliff’s δ ≈ 0.27, with a 95% CI spanning zero). **D** ECDFs for 3′-UTR ΔUTR values, consistent with the non-significant group difference

DRS-defined transcript-end sites (TES) enabled mapping of the experimentally validated UAAA polyadenylation-signal upstream of TES and quantification of poly(A)-tail lengths in intron-containing genes and genes without curated introns [20, 21, 23]. The revised analysis revealed enrichment of the UAAA signal approximately 10–30 nt upstream of TES, and poly(A)-tail length distributions for intron-containing genes centered at ~ 60–80 nt (Tables S1 and S2). Separately, using StringTie v2.2.1 mixed-mode assemblies (Illumina, ONT DRS, and ONT cDNA; anchored with -G to the TrichDB G3 2022 reference), the representative transcription start site (TSS) was defined as the 5′ end of the highest-TPM isoform per gene, with changes within ± 10 nt considered unchanged. Across all comparable genes (*n* = 28,929), 23.2% (6,723/28,929) showed 5′-UTR extension (median + 68 nt, IQR 21–193), 17.7% (5,117/28,929) showed shortening (median − 43 nt, IQR − 175 to − 19), and 59.1% were unchanged. Applying expression thresholds increased concordance while preserving the overall extension bias: at TPM ≥ 1 (*n* = 20,701), extension/shortening/unchanged were 21.6%/7.8%/70.6% (medians + 52/ − 21 nt), and at TPM ≥ 5 (*n* = 11,358) they were 18.6%/2.7%/78.7% (medians + 24/ − 19 nt). Direct comparison between intron-containing genes and genes without curated introns revealed a significant 5′-end right shift in intron-containing genes (Mann–Whitney U *P* = 0.0052; Cliff’s δ ≈ 0.25; BCa 95% CI 0.10–0.40; intron *n* = 40, non-intron *n* = 28,889), whereas 3′-UTR differences were not significant at the current sequencing depth (Mann–Whitney U *P* = 0.125; Cliff’s δ ≈ 0.27, with a 95% CI spanning zero; intron *n* = 11, non-intron *n* = 11,346) (Fig. 4). Together, these results indicate that long-read transcriptomics uncovers upstream UTR regions that are under-represented in the reference annotation, with a more pronounced 5′-end extension signal in intron-containing genes.

### Reconciliation with the short-read catalog

Callejas-Hernández et al. recently reported 63 “active” and 34 “inactive” intron sequences in *T. vaginalis* using short-read RNA-seq on a chromosome-scale assembly, highlighting an exceptionally short 23-nt intron, an apparent expansion of UTR-localized introns, and partial conservation in *T. stableri* [19]. Leveraging Oxford Nanopore DRS, ONT cDNA long reads, and Illumina RNA-seq, we re-evaluated all 63 reported active loci on the NYU_TvagG3_2 assembly and updated their genomic coordinates and supporting evidence tiers. Of these, 56 of 63 mapped uniquely and were supported by long-read splice-junction evidence. Five loci required correction, including two false positives, two coordinate misannotations, and one underlying gene-model sequence error, while two loci lacked detectable expression in both our datasets and public DRS replicates but remained PCR-positive at the genomic level (Table S3; Figs. 1 and 2). In addition, long-read transcriptomics identified two bona fide introns not captured in the original short-read active-intron catalog, each supported by multiple full-length reads and PCR validation. Relative to the current NYU_TvagG3_2 reference annotation, the curated set includes three previously unrecognized introns; relative to the Callejas-Hernández et al. active-intron catalog, two introns were not captured by the original short-read pipeline. For all loci, we provide reconciled coordinates, read-level support, PCR status, and, where available, DRS-derived poly(A)-tail lengths and UTR boundary annotations (Tables S1 and S2).

### Proteomic evidence supports translation of re-annotated transcripts

To verify whether the newly identified intron-containing transcripts are translated into stable proteins, we performed LC–MS/MS analysis using a custom protein database constructed from our 17 validated long-read gene models. Despite the typically low abundance of splicing factors and signaling proteins, we identified high-confidence unique peptides for two of the candidates: TVAGG3_CG07 (annotated as a CAMK-family kinase) and TVAGG3_CG08 (Table S4).

For TVAGG3_CG07, a unique peptide (IADFGFAR) was detected (6 PSMs), supporting expression of the re-annotated coding sequence. Similarly, TVAGG3_CG08, which contains a newly identified 5′-UTR intron, yielded a unique peptide match (NSYELTATLLMFLNMLAVSMR with methionine oxidations) with a high-confidence score. Detection of peptides from these loci provides orthogonal evidence that at least a subset of transcripts defined by our long-read pipeline can represent processed, translated products rather than degradation artifacts. The absence of peptides for the remaining 15 candidates is likely attributable to the limited dynamic range of shotgun proteomics and the potentially low expression levels of these specific isoforms under standard axenic culture conditions.

## Discussion

This study applies an integrated long-read transcriptomic framework—combining Oxford Nanopore direct RNA sequencing (DRS) and ONT cDNA reads with Illumina short-read RNA-seq, supported by PCR and Sanger validation—to refine splicing and untranslated region (UTR) annotations in *Trichomonas vaginalis*. We expand the catalog of validated introns from 31 to 48 by adding 17 newly confirmed events (11 Type A and 6 Type B). In addition, long-read evidence enables correction of multiple gene models, including merging of previously split loci, precise delineation of 5′ and 3′ UTR boundaries, and single-molecule estimation of poly(A) tail lengths. In a genome dominated by repetitive elements and large gene families, this hybrid strategy substantially reduces annotation artifacts and false-positive intron calls common in short-read-only approaches, providing a more accurate structural and functional reference for downstream analyses.

Our results are broadly concordant with those of Callejas-Hernandez et al. [19] in that we similarly observe an expansion of UTR-localized introns and the presence of exceptionally short introns in *T. vaginalis*. Notably, the ultrashort 23-nt intron reported in their study is fully supported by our data. Multiple long-read molecules (both DRS and ONT cDNA) span this 23-nt region with clear GT–AG splice junctions, and representative cases were independently validated by PCR–Sanger sequencing, confirming that such minimal introns represent genuine biological features rather than alignment artifacts. Beyond confirmation, long-read data enabled empirical refinement of specific loci and gene models. Among paralogous candidates, TVAGG3_0931505/TVAGG3_0930320 are strongly supported by long-read evidence, whereas TVAGG3_0105790/TVAGG3_0998030 remain unresolved at current sequencing depth. We also identified a previously unannotated intron in TVAGG3_CG12 (TVAGG3_0480470), leading to updated UTR boundary definitions. Importantly, TVAGG3_CG14 (TVAGG3_0143860) was previously annotated as carrying a 5′-UTR intron [19]; however, our long-read mapping demonstrates that the transcript spans both TVAGG3_0143860 and TVAGG3_0143870. Following model merging, the intron is correctly repositioned within the coding sequence (CDS). This correction is functionally significant, as introns located in UTRs versus CDS regions impose distinct constraints on transcript processing and protein-coding potential.

**Fig. 5:**
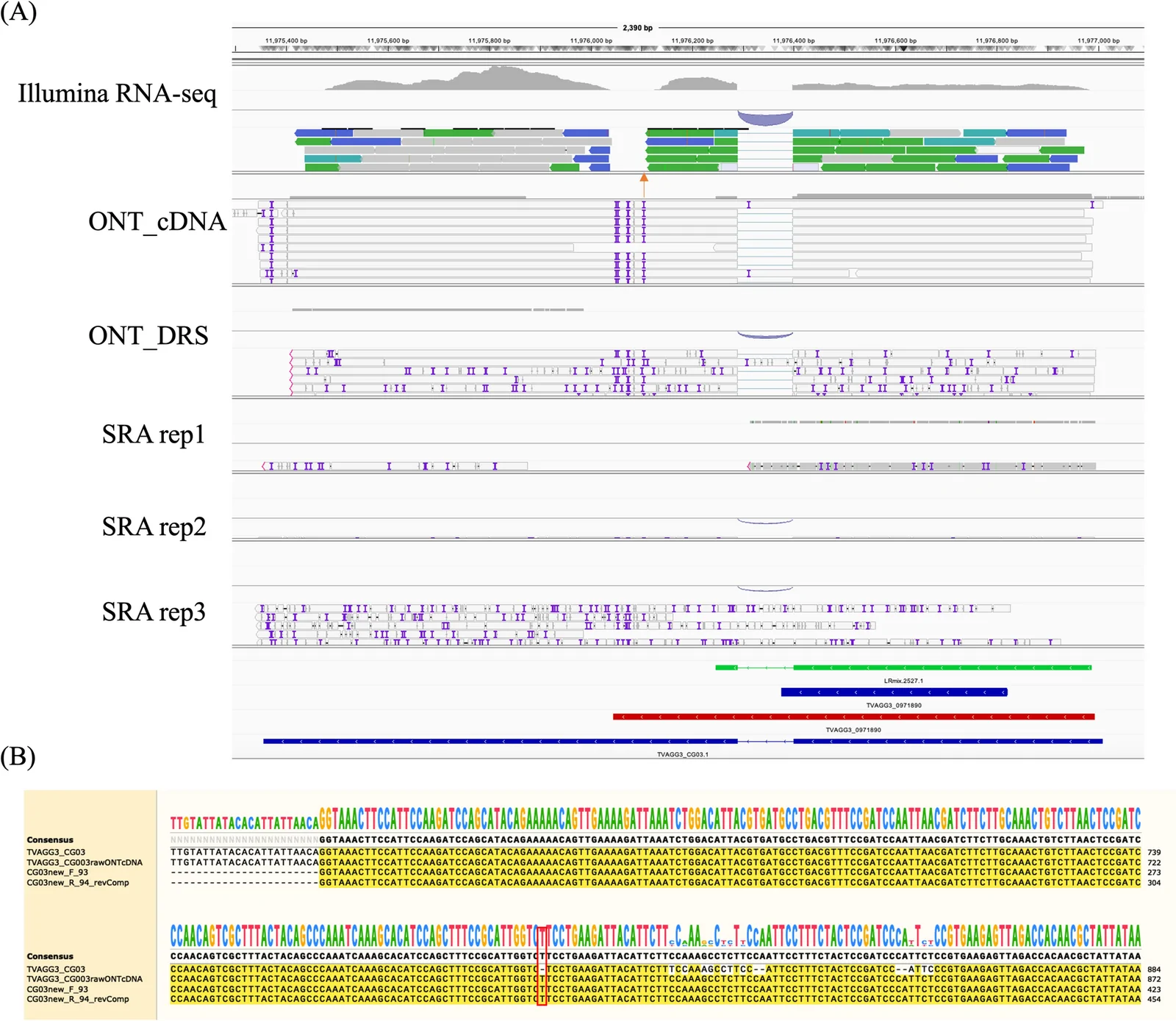
Long-read sequencing and Sanger validation correct a reference genome error at the TVAGG3_CG03 locus. **A** Integrative Genomics Viewer (IGV) snapshots displaying alignments from Illumina RNA-seq, ONT cDNA, ONT DRS, and public SRA datasets, all consistently supporting a conserved intronic gap. The orange arrow indicates a coverage breakpoint in the Illumina short reads, corresponding to insertion/deletion (indel) markers shown in purple in the ONT long reads. The bottom annotation tracks display gene models from different sources: a hybrid transcriptome prediction generated from combined Illumina and ONT data (green, LRmix.2527.1), the current reference genome annotation (blue, TVAGG3_0971890), the annotation from Callejas-Hernández et al. [19] (red, TVAGG3_0971890), and the corrected continuous gene model from this study (bottom blue, TVAGG3_CG03.1). **B** Multiple sequence alignment validating the genomic correction. Because this locus is located on the reverse strand, the red box highlights the indel discrepancy corresponding to the orange-arrow position in **A**, with additional indels visible further downstream. The alignment compares the genomic sequence extracted from the reference coordinates (TVAGG3_CG03), a representative ONT cDNA read (TVAGG3_CG03rawONTcDNA), and our Sanger sequencing results (forward, CG03new_F_93; reverse complement, CG03new_R_94_revComp). The empirical data correct reference sequence errors, restore the proper reading frame, and support a single continuous gene model

These refinements highlight important methodological considerations for repeat-rich protist genomes, such as *T. vaginalis*. Short-read data are particularly susceptible to distant mis-mapping and artifactual splice-junction calls spanning kilobase-to-megabase-scale distances, which can erroneously appear as multi-intron transcripts or gene fusion events. In our dataset, such predictions consistently failed PCR validation and were subsequently rejected upon inspection of long-read evidence. Figure 5 illustrates a definitive example (TVAGG3_CG03) in which long-read sequencing resolves an underlying reference genome error. When open reading frames (ORFs) were inferred solely from published genomic coordinates, two separate coding segments were predicted. In contrast, a proovread-corrected ONT cDNA read spans the full locus as a single continuous ORF and matches the Sanger sequence exactly, demonstrating that the apparent "split ORF" resulted from reference base-calling or assembly artifacts rather than true biological variation. Alignment strategy also critically affects the interpretation of complex loci. For long-read mapping, we used minimap2 in splice-aware mode (-ax splice -k14 -G 10000 -L), which balances sensitivity to true splice junctions with suppression of spurious long-range alignments in repeat-dense regions.

Beyond intron identification, DRS enabled quantification of poly(A)-tail lengths and mapping of the spatial relationship between UAAA polyadenylation-signal (PAS) positions and transcript-end sites (TES), consistent with prior functional studies establishing UAAA as the functional polyadenylation signal in *T. vaginalis* [20, 21, 23]. We observed enrichment of the UAAA motif approximately 10–30 nt upstream of TES, and poly(A)-tail length distributions centered around 60–80 nt for intron-containing genes, suggesting a potential association between intron architecture and 3′-end processing features. While causality cannot be inferred from these data alone, the resulting resource provides a framework for future studies investigating whether UTR introns influence cleavage and polyadenylation efficiency. At the 5′ end, mixed transcriptome assemblies recover substantial upstream UTR sequences absent from the current reference annotation, leading to a systematic extension bias. At the 3′ end, long-read-defined TES positions refine gene boundaries and in several cases, merge previously fragmented gene models into single, continuous transcriptional units. Collectively, these improvements yield more accurate gene models, facilitating functional characterization, experimental validation, and diagnostic assay design.

While this study robustly validated splice junctions for all 17 candidates via PCR and Sanger sequencing, proteomic evidence was obtained for a subset (TVAGG3_CG07 and TVAGG3_CG08). This outcome is not unexpected given the intrinsic limitations of detecting low-abundance signaling proteins in complex whole-cell lysates using untargeted shotgun proteomics. Importantly, the identification of unique peptides for these genes provides orthogonal evidence that the ORFs derived from our long-read pipeline are actively expressed and translated. Future studies employing targeted proteomics (e.g., PRM) or epitope-tagging strategies will be required to comprehensively characterize translation products across the remaining candidates. Additional limitations include incomplete long-read coverage for lowly expressed or condition-specific transcripts and residual base-calling inaccuracies in DRS data, particularly in homopolymeric regions. Nevertheless, our integrated long-read framework demonstrates that it can simultaneously resolve single-molecule poly(A)-tail lengths, UAAA–TES spatial organization, high-resolution UTR boundaries, and empirically corrected gene models. Together, these advances reconcile inconsistencies in previous short-read annotations with experimentally grounded evidence, confirm the existence of ultrashort 23-nt introns, and provide a high-resolution foundation for studying transcript processing and gene regulation in *T. vaginalis*. The continued identification of novel intron-bearing loci, despite the genome's compact, repeat-rich nature, further underscores the functional complexity of its RNA processing machinery. Notably, recent work has suggested unusual spliceosomal plasticity in this divergent protozoan, including the capacity for trans-splicing of split introns [38]. Together with our multi-omics validation of 17 intronic loci and corrected gene models, these findings support the view that the spliceosomal system in *T. vaginalis* exhibits greater functional flexibility and evolutionary divergence than previously appreciated.

## Conclusions

By integrating long-read transcriptomics with targeted proteomic and Sanger sequencing validation, this study reconciles and refines the existing short-read intron catalog for* Trichomonas vaginalis*. Beyond expanding the set of validated introns, we refined transcript-end annotations using DRS-supported TES mapping, the experimentally validated UAAA polyadenylation signal, and long-read-derived UTR boundary information while also demonstrating the critical role of long reads in resolving structural errors in the reference genome. These multi-omics-supported coordinates provide a reliable framework for functional genomics, studies of transcript processing, and the development of precise diagnostic assays.

## Supplementary Information

Supplementary material 1: Figure S1. PCR validation of annotated intron-containing genes in* Trichomonas vaginalis*. Agarose gel electrophoresis of PCR amplicons generated from genomic DNA (gDNA) and cDNA templates. Each panel corresponds to one of the 31 reference intron-containing genes, with expected amplicon sizes (gDNA | cDNA, in base pairs) indicated below the gene IDs. For each gene, the left lane shows the gDNA amplicon and the right lane shows the cDNA amplicon; a synthetic DNA ladder is provided on the far right for size reference. For most candidates, bands of the expected sizes confirm the genomic presence of the annotated introns and successful splicing of the transcripts. The absence of cDNA or gDNA amplicons in specific panels, such as TVAGG3_0536040 and TVAGG3_0511450, corresponds to genes lacking detectable expression or amplification under the assayed conditions, consistent with the transcriptomic evidence summarized in Supplementary Table S1.

Supplementary material 2: Figure S2. Transcriptomic coverage profiles of the 31 reference intron-containing genes. Integrative Genomics Viewer (IGV) snapshots display read alignments across the 31 annotated loci in *Trichomonas vaginalis*. For each gene panel, the alignment tracks from top to bottom represent Illumina short reads (SR), Nanopore cDNA reads (cDNA), and Nanopore direct RNA reads (DRS). The bottom tracks denote the reference gene models. Split-read alignments and coverage gaps define intron boundaries across multiple datasets. In concordance with PCR validation (Supplementary Figure S1), specific loci, such as TVAGG3_0511450 and TVAGG3_0536040, exhibit minimal or no detectable transcriptomic coverage, consistent with the lack of cDNA amplification.

Supplementary material 3: Figure S3. Transcriptomic coverage profiles of the 17 additional validated intron-containing genes in* Trichomonas vaginalis*. Integrative Genomics Viewer (IGV) snapshots display read alignments across the 17 additional validated or structurally corrected loci (TVAGG3_CG01 to TVAGG3_CG17). For each gene panel, the alignment tracks from top to bottom represent Illumina short reads (SR), Nanopore cDNA reads (cDNA), and Nanopore direct RNA reads (DRS). Split-read alignments and sharp drops in read coverage support intron boundaries across multiple sequencing datasets. These visual findings corroborate the experimental and transcriptomic evidence summarized in Supplementary Table S2.

Supplementary material 4: Table S1. Overview of 31 annotated intron-containing genes in the *Trichomonas vaginalis* G3 2022 reference genome. This table lists each gene’s basic information, intron type, and exon coordinates. Multiple lines of experimental and transcriptomic evidence are indicated by check marks (✓, presence) and crosses (✗, absence), including gel electrophoresis (Gel), Illumina short reads (SR), Nanopore cDNA (ONT cDNA), Nanopore direct RNA (ONT DRS), and public SRA datasets. Quantitative poly(A)-tail features derived from DRS data are provided where available, including mean and median poly(A)-tail lengths in nucleotides with associated interquartile ranges (IQR), as well as the nearest upstream UAAA/TAAA motif, median UAAA-to-TES distance, and supporting read counts where available. Signed TES offsets from annotated 3′ ends are reported separately from UAAA-to-TES distances.

Supplementary material 5: Table S2. Summary of 17 additional validated intron-containing genes in *Trichomonas vaginalis* G3 2022 assembly. This table lists the 17 additional intron-containing genes that were validated or structurally corrected in the present study. For each gene, the following information is provided: assigned gene name, corresponding reference gene name, functional description, chromosome, strand orientation, intron type (A or B), and exact genomic coordinates for the intron and flanking exons. Validation evidence is indicated by check marks (✓, presence) and crosses (✗, absence) across multiple parameters and platforms, including successful primer design, gel electrophoresis (Gel check), Illumina short reads (SR), Nanopore cDNA (ONT cDNA), Nanopore direct RNA (ONT DRS), and public SRA datasets. Quantitative poly(A)-tail features derived from DRS data are provided where available, including mean and median poly(A)-tail lengths in nucleotides with associated interquartile ranges (IQR), as well as the nearest upstream UAAA/TAAA motif, median UAAA-to-TES distance, and supporting read counts where available. Signed TES offsets from annotated 3′ ends are reported separately from UAAA-to-TES distances.

Supplementary material 6: Table S3. Long-read re-evaluation of previously reported introns and one additional locus in *Trichomonas vaginalis*. This table cross-references 64 intron-bearing entries, including the 63 active introns reported by Callejas-Hernández et al. [19] and one additional intron-containing locus validated in the present study. Original 2007 reference genome IDs (TVAG_) and updated G3 2022 IDs (TVAGG3_) are provided where applicable. For each locus, we summarize strand orientation, intron type (A or B, based on splice-site motifs), curatorial status, functional annotation, and salient remarks. Validation evidence is indicated by check marks (✓, presence) and crosses (✗, absence) across multiple platforms, including gel electrophoresis (Gel), Illumina short reads (SR), Nanopore cDNA (ONT cDNA), and Nanopore direct RNA (ONT DRS). Quantitative poly(A)-tail features estimated from DRS reads are provided where available, including mean and median poly(A)-tail lengths, interquartile range (IQR), the nearest upstream UAAA/TAAA motif, median UAAA-to-TES distance, and supporting read counts. Signed TES offsets from annotated 3′ ends are labeled separately from UAAA-to-TES distances. This compilation enables rapid assessment of concordance, structural corrections, and additional validated introns relative to historical and recent annotations.

Supplementary material 7: Table S4. Proteomic identification of newly validated intron-containing gene products. Peptide-spectrum match (PSM) details are shown for unique peptides mapping to TVAGG3_CG07 and TVAGG3_CG08. The table includes protein accession, peptide sequences, modifications, PSM counts, mass accuracy (DeltaM), and search engine confidence scores, providing orthogonal evidence supporting translation of these re-annotated transcripts.

Supplementary material 8: Table S5. Primer sequences used for amplification and validation of the 48 confirmed introns in *Trichomonas vaginalis*. This table lists the forward and reverse primer sequences (5′–3′) designed for experimental validation of all 48 confirmed intron-containing loci investigated in this study, comprising 31 historically annotated introns and 17 additional validated introns. Loci are categorized by curatorial status and intron type, including additional validated, Type A, Type B, and motif-unassigned introns. Expected amplicon sizes for both genomic DNA (gDNA) and spliced transcripts (cDNA) are provided in base pairs (bp), corresponding to the gel electrophoresis results shown in Supplementary Figure S1 and related validation experiments.

## Data Availability

The datasets generated and/or analyzed during the current study are available in the NCBI Sequence Read Archive (SRA) repository under BioProject accession number PRJNA1311745. Additional data generated or analyzed during this study are included in this published article and its supplementary information files.

## Abbreviations

ATCC: American Type Culture Collection
BCa: Bias-corrected and accelerated
CAMK: Calmodulin-dependent protein kinase
cDNA: Complementary DNA
CDS: Coding sequence
DRS: Direct RNA sequencing
ECDF: Empirical cumulative distribution function
FDR: False discovery rate
FISH: Fluorescence in situ hybridization
gDNA: Genomic DNA
IGV: Integrative Genomics Viewer
IQR: Interquartile range
LC–MS/MS: Liquid chromatography–tandem mass spectrometry
NCBI: National Center for Biotechnology Information
NTC: No-template control
ONT: Oxford Nanopore Technologies
ORF: Open reading frame
PAS: Polyadenylation signal
PCR: Polymerase chain reaction
PRM: Parallel reaction monitoring
PSM: Peptide–spectrum match
RNA-seq: RNA sequencing
SRA: Sequence Read Archive
STI: Sexually transmitted infection
TE: Transposable element
TES: Transcript-end site
TPM: Transcripts per million
TSS: Transcription start site
UTR: Untranslated region
YI-S: Yeast extract–iron–serum
